# Myocardial Bridging: Review on the Role of Coronary Computed Tomography Angiography

**DOI:** 10.3390/jcm12185949

**Published:** 2023-09-13

**Authors:** Chiara Rovera, Claudio Moretti, Francesca Bisanti, Giulia De Zan, Marco Guglielmo

**Affiliations:** 1 Department of Cardiology, Civic Hospital of Chivasso, 10034 Chivasso, Italy; crovera@aslto4.piemonte.it (C.R.); cmoretti@aslto4.piemonte.it (C.M.); 2 Department of Radiology, Civic Hospital of Chivasso, 10034 Chivasso, Italy; fbisanti@aslto4.piemonte.it; 3 Department of Cardiology, Division of Heart and Lungs, Utrecht University Medical Center, 3584 CX Utrecht, The Netherlands; g.dezan@umcutrecht.nl; 4 Department of Translational Medicine, University of Easter Piedmont, Maggiore della Carita’ Hospital, 28100 Novara, Italy

**Keywords:** myocardial bridging, coronary vessel anomalies, congenital heart defects, computed tomography angiography, diagnostic imaging

## Abstract

Myocardial bridging (MB) is a congenital coronary anomaly in which a segment of a coronary artery, most frequently the left anterior descending artery, deviates from its epicardial route by passing through the myocardium. The advent of cardiac computed tomography angiography (CCTA), equipped with its multiplane and three-dimensional functionalities, has notably enhanced the ability to identify MBs. Furthermore, novel post-processing methods have recently emerged to extract functional insights from anatomical evaluations. MB is generally considered a benign entity with very good survival rates; however, there is an increasing volume of evidence that certain MB characteristics may be associated with cardiovascular morbidity. This review is intended to depict the diagnostic and prognostic role of CCTA in the MB context.

## 1. Introduction

Myocardial bridging (MB) is an anatomical entity whereby a portion of the epicardial coronary artery takes an intramyocardial route. 

The layer of muscle that covers the artery is known as a “myocardial bridge,” and the artery that traverses through the myocardium is labeled as a “tunneled artery” [1].

This phenomenon was first mentioned in 1737 by Reyman in autopsy series [2], but Geiringer was the first to perform detailed morphological studies in 1951 [3]. 

MB can occur in any of the coronary arteries, but the most common artery affected is the left anterior descending artery (LAD, 76.9%), with its mid segment being the most frequent site of bridging (46.7%), followed by the distal LAD [4].

The prevalence of MB varies widely depending on the diagnostic modality applied. In autopsy series, the prevalence varies from 20% to 85% [4], which is substantially higher than that in angiographic studies (0.5–3%) [1]. With the advent of cardiac computed tomography angiography (CCTA), there has been a paradigm shift in the modality preferred for identifying MB; such identification is facilitated using the three-dimensional and multiplanar reformats available with this modality. CCTA is a non-invasive technique with the ability to detect the length and depth of the MB, MB morphology, and compression produced by the MB [5,6]. However, even with CCTA, the prevalence of MB varies considerably from 3.5% to 58% [7,8,9,10]. The variance in the frequency of reported MB on CCTA most likely reflects the evolution in CCTA technology over the past decades. Furthermore, it is unknown whether environmental and geographical factors also play a contributory role.

In most patients, MB is an incidental finding associated with an excellent survival rate of 97% at 5 years [11]. However, it is not entirely a benign entity. There have been reported associations with myocardial ischemia [12], myocardial infarction [13,14,15,16,17], arrhythmia [18], and sudden death [19]. The clinical significance of a MB appears to be related to (1) the anatomic properties of the tunneled segment of the coronary artery, (2) the presence of associated myocardial ischemia, and (3) the presence of proximal and distal atherosclerotic disease [20].

**Figure 1:**
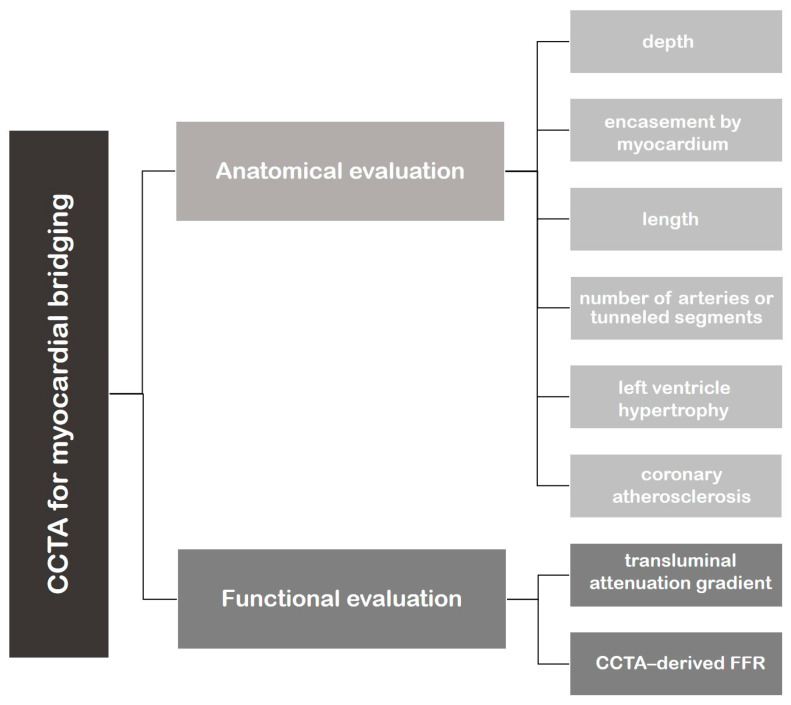
Flowchart representing the application of cardiac computed tomography angiography (CCTA) in the evaluation of myocardial bridging.

In the MB setting, CCTA allows for anatomical visualization of both the coronary artery lumen and the myocardium and, more recently, the functional assessment for evaluating the clinical relevance of MB (Figure 1). 

This review is intended to illustrate the feasibility and potential value of CCTA in MB.

## 2. CCTA Imaging Protocol

CCTA images for the detection of MB are obtained using the standard protocol [21,22].

The target coronary attenuation value is 350–400 Hounsfield units (HU), achieved through an iodinated contrast medium volume of 245–370 mgI/kg administered intravenously into an antecubital vein at a flow rate of 4–5 mL/s for 10–20 s. A 50–60 mL saline flush is used at the same injection rate as the contrast material. A bolus-tracking method is employed with monitoring placed in the ascending aorta. 

A tube potential of 100 peak kilovoltage (kVp) is applied in patients with body weight ≤ 100 kg or BMI < 30 kg/mq and an automated current adjustment mode is utilized. The scan range covers the entire heart, starting from the tracheal bifurcation to the diaphragm with craniocaudal scan direction during the middle inspiratory breath-hold.

A high-pitch acquisition can be considered in patients with stable sinus rhythm and a heart rate of less than 60 bpm and a body weight ≤100 kg (very low radiation dose); a prospective ECG-gated acquisition is performed in situations with a regular rhythm, where high-pitch or high-volume scans are not available or contraindicated; retrospective ECG-gated acquisition should be considered in patients with an irregular rhythm or high heart rate. 

With the introduction of the 320-slice multi-slice CT, scanning of the entire heart has become possible in a single heartbeat with a very low dose of radiation [23]. 

If the heartbeat is greater than 65 bpm, intravenous β-blockers are given before CCTA; furthermore, after ruling out hypotension, a commonly used regimen of 400–800 μg sublingual nitroglycerin is administered 5 min before the exam.

The image reconstructions are done using 10 phases in 10% of each RR interval. Images are reconstructed with a slice thickness of <1 mm, a slice increment of 50% of the slice width, an image matrix of 512 × 512, and a field of view (FOV) of 200–250 mm. 

A diastolic and systolic dataset with the best image quality is selected. Images are restructured using multiple post-processing methods. Transverse source images, multiplanar reformations (MPR), curved multiplanar reformations, and maximum intensity projection images are used for evaluating the intramyocardial course. 

Whole-heart volume-rendered images of the heart are also reconstructed and rotated to best display the coronary arterial anatomy. 

## 3. Role of CCTA: Anatomical Evaluation

CCTA has significantly improved the anatomical characterization of MB. Its strength lies in its high spatial resolution. 

**Figure 2:**
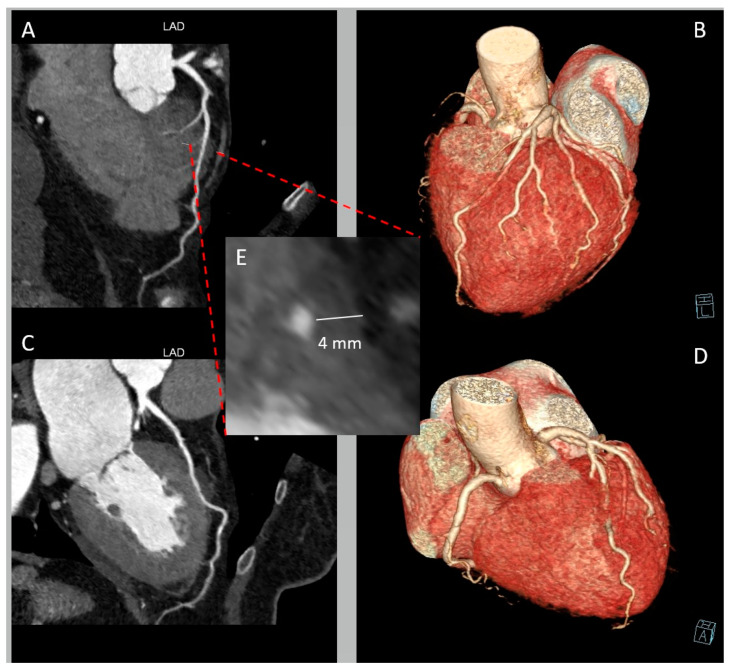
Deep myocardial bridging (MB) of left anterior descending artery (LAD) in a 65-year-old woman presenting with chest pain and negative V1-V5 T waves. (**A**) Curved multi-planar reformation of LAD providing a depiction of MB in the mid segment. (**B**) Whole-heart volume-rendered image showing the “tunneled” LAD. (**C**) Curved multi-planar reformation of LAD of the same patient in systolic phase. (**D**) Whole-heart volume-rendered image in systolic phase. (**E**) Cross-sectional image displaying LAD-MB depth.

In terms of anatomy, LAD-MBs can exhibit variations in depth, categorized as superficial (>1 to 2 mm), deep (≥2 mm), and very deep (≥5 mm) [10] (Figure 2). The degree of systolic compression of the artery is linked to the depth of the tunneled artery, although it is not the sole determining factor. When considering treatment, particularly surgical intervention, the depth of the MB also holds significance. 

**Figure 3:**
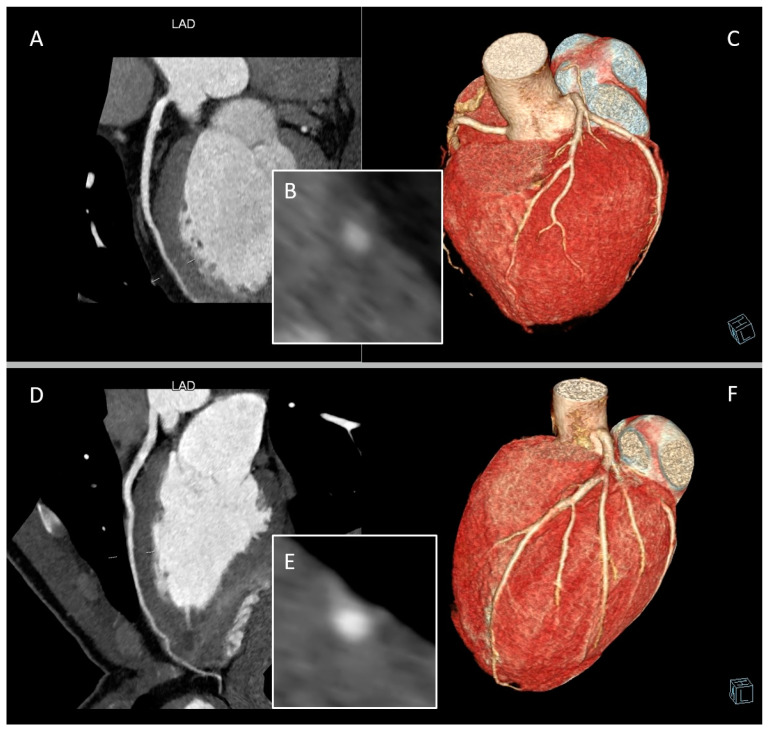
Complete and partial myocardial bridging (MB) of left anterior descending artery (LAD). Panels (**A**–**C**) are from a 54-year-old man classified as intermediate cardiovascular risk and symptomatic for chest discomfort during exertion. Panels (**D**)–(**F**) are from an 18-year-old athlete with altered cardiac repolarization during exercise. (**A**) Curved multi-planar reformation of LAD providing a depiction of a superficial complete MB. (**B**) Cross-sectional image displaying LAD-MB depth and the entire encasement by myocardium. (**C**) Whole-heart volume-rendered image of the same patient. (**D**) Curved multi-planar reformation of LAD providing a depiction of a partial MB. (**E**) Cross-sectional image displaying LAD-MB partial encasement. (**F**) Whole-heart volume-rendered image of the same patient.

CCTA can describe muscle bridging as being partial or complete, depending on the extent to which the coronary artery segment with bridging is surrounded by myocardium. A partial MB is one in which >75% but <100% of the coronary artery is encased by myocardium, and a complete bridge is one that is entirely encased by myocardium [10] (Figure 3).

**Figure 4:**
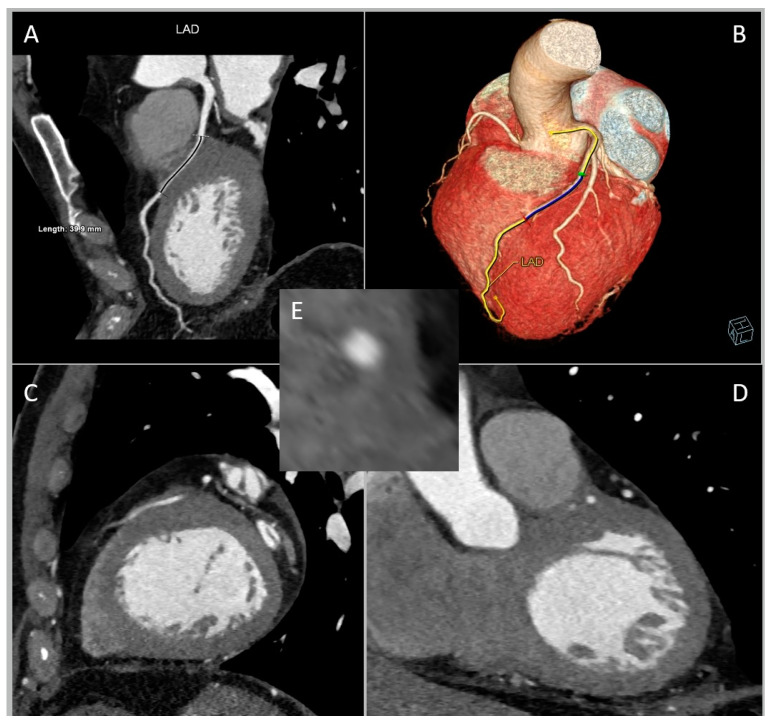
Long myocardial bridging (MB) of left anterior descending artery (LAD) in a 69-year-old man referred for chest pain in arterial hypertension, dyslipidemia, and ex-smoking status. (**A**) Curved multi-planar reformation of LAD. (**B**) Whole-heart volume-rendered image. (**C**) Sagittal plane. (**D**) Coronal plane. (**E**) Cross-sectional image.

Third, MB is classifiable into short (<25 mm) or long (≥25 mm) according to the length of the tunneled segment (Figure 4).

The length of the encasement appears to impact the dynamic compression of both the trapped LAD segment and the septal branches originating from or near the affected LAD segment [24]. One repercussion is the occurrence of “branch steal”: when blood moves through the tunneled artery during late systole/early diastole, it traverses a narrowed section that raises fluid speed, leading to reduced perfusion pressure at the entrance of the septal branch due to the Venturi effect. The existence of “branch steal” in MB is substantiated using the finding that mild-to-moderate MBs more frequently lead to septal ischemia compared to ischemia in the distal myocardium. 

Since both the length and depth of the MB are closely related to systolic compression, their synergy may generate contractile force exerted by the MB. To assess the mass volume of the MB muscle, the value of MB depth (in millimetres) multiplied by MB length (in millimetres) is defined as the MB muscle index (MMI) [25].

**Figure 5:**
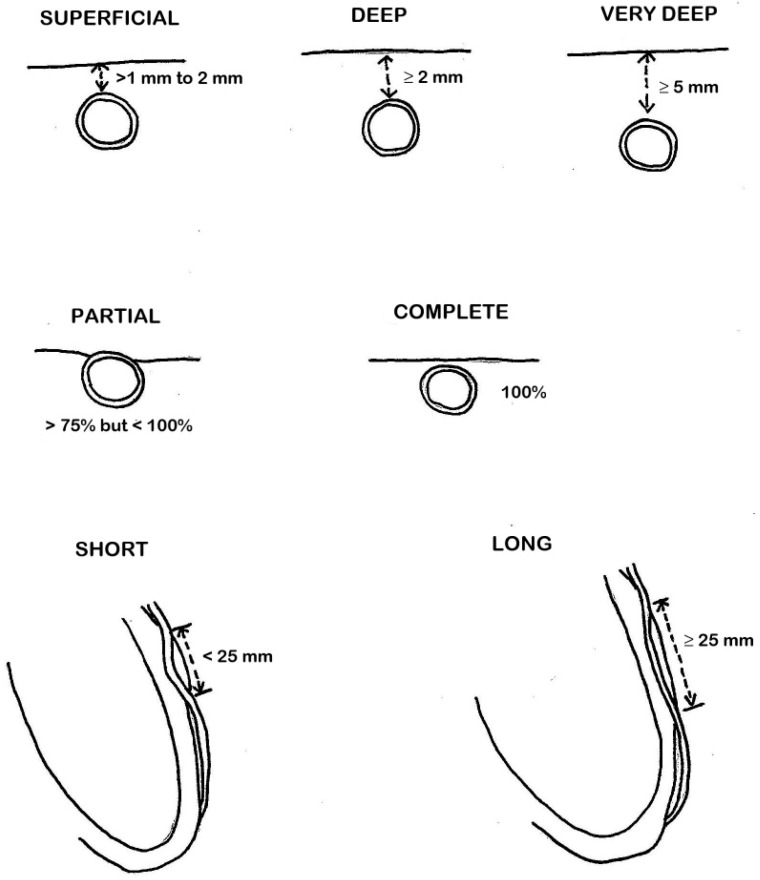
Schematic representation of anatomical classifications of myocardial bridging (MB).

The major anatomical MB classifications are schematically outlined in Figure 5.

When conducting CCTA, it is crucial to consider any additional significant anatomical characteristics of the LAD-MB, such as the concurrent number of arteries or tunneled segments, as well as the degree of systolic diameter reduction or kinking.

MBs that develop into symptomatic disease often occur in a complex interplay also related to left ventricle hypertrophy and the presence of coronary atherosclerosis [26]. 

Even in this field, CCTA can be of great help for anatomical evaluation. 

Regarding left ventricular hypertrophy, information on left ventricular mass is readily available for analysis without additional testing [27] and is derived using semi-automated delineation with manual correction of the endocardial and epicardial borders at end-diastole, with exclusion of the papillary muscles.

**Figure 6:**
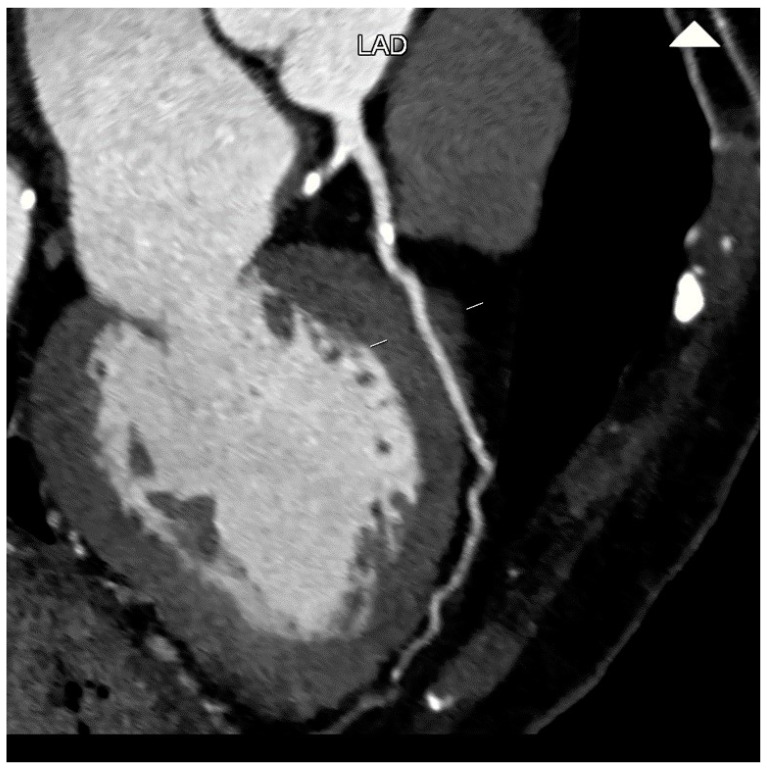
Atherosclerotic plaque occurring proximal to the myocardial bridging (MB) of left anterior descending artery (LAD) in curved multi-planar reformation. This CCTA image belongs to an 82-year-old woman suffering from arterial hypertension and complaining of chest pain during exertion.

Furthermore, the development of atherosclerotic coronary disease has been associated with MB. The systolic compression of the tunneled artery produces retrograde flow proximal to the MB [28]. This results in abrupt breakage of the propagating antegrade systolic wave and creates an area of low wall shear stress that has been posited to explain the development of atherosclerotic plaque immediately proximal to the MB [26]. The presence of atherosclerotic plaque located proximal to the MB leads to reduced pressure downstream, which worsens the ischemia induced by the MB. CCTA is very reliable in the quantification and characterization of atherosclerotic burden and location (Figure 6). 

Nonetheless, these altered biomechanical forces might also be accountable for certain non-atherosclerotic complications linked to the MB. It is plausible that the elevated compressive stress exerted by the MB onto the tunneled artery could induce intimal damage, potentially evolving into dissection [29].

Another frequently observed characteristic of MB is its relatively low occurrence of plaque within the tunneled artery segment. This phenomenon can be elucidated through several observations: Firstly, MB leads to the separation of the coronary artery from the perivascular adipose tissue in the epicardium, which is tied to proinflammatory signaling pathways [30]. Secondly, imaging with optical coherence tomography has noted the absence of adventitial vasa vasorum, which typically function as conduits for the diffusion of inflammatory cells and cytokines from the perivascular adipose tissue [31]. Thirdly, the compression of the tunneled artery might enhance lymphatic drainage [32]. Lastly, the tunneled segment experiences heightened or physiologically significant wall shear stress due to elevated velocities, a factor associated with atheroprotective pathways in vitro [33].

Distally, due to increased endothelial shear stress, decreased blood flow and volume, atherosclerosis is underdeveloped and a decrease in luminal diameter (secondary coronary hypoplasia) is often noticeable.

## 4. Role of CCTA: Functional Evaluation

CCTA can not only serve as a valuable non-invasive test for the anatomical definition of a MB, but can also provide insight into the potential hemodynamic significance of the bridge in patients with recurrent symptoms of typical angina without obstructive coronary artery disease. 

Categorized by the degree of lumen constriction during the systolic phase in CCTA, individuals with MB can be sorted into three categories: those lacking systolic compression and without luminal narrowing, those with <50% systolic compression, and those with systolic compression leading to ≥50% luminal stenosis. Nonetheless, it has been acknowledged that dynamic compression is not solely linked to the systolic phase; it also continues into the mid-to-late diastole [34].

Moreover, a patient undergoing CCTA is usually administered a combination of beta-blockers, calcium antagonists, and/or ivabradine along with nitroglycerin prior to the scan. This step is necessary to enhance image quality by extending the diastolic phase and promoting coronary vasodilation. Nevertheless, these medications have the potential to mask the clinical importance of MBs, thereby constituting a constraint in the study of MBs through CCTA. Employing dobutamine and/or exercise to induce the physiological state in which MBs are most likely to elicit symptoms thus poses a notable technical obstacle. Nonetheless, last-generation CT scanners have recently been used to perform CCTA stress perfusion. 

In more recent times, novel post-processing methods have emerged to extract functional insights from the anatomical evaluation conducted through CCTA.

Two of these techniques include the Transluminal Attenuation Gradient (TAG) and the CCTA-derived Fractional Flow Reserve (FFR) [35].

The former entails the linear regression coefficient between luminal attenuation and the axial distance from the coronary ostium [36,37]. 

TAG is characterized as the transluminal contrast intensity gradient in CCTA from the proximal to the distal regions of vessels. For each targeted vessel, cross-sectional images are reconstructed perpendicular to its centerline. Hounsfield units (HU) determine the TAG and are measured at 5-mm intervals, ranging from the ostium to the point where the lumen area falls below 2 mmq. This attenuation gradient is established by assessing the HU change over a 10-mm span of the coronary artery. It is defined using the linear regression coefficient between the intraluminal attenuation in HU and the distance from the vessel ostium in millimeters.

The TAG demonstrates a direct linear relationship with the degree of intracoronary transluminal stenosis [38]. Importantly, the systolic compression can be excluded from the evaluation of the intracoronary TAG derived from the diastolic phase. Furthermore, using an optimal cutoff value (≤–18.8 HU/10 mm) as a benchmark, MB cases with systolic compression of ≥50% can be distinguished from those with mild systolic compressions [39].

The CCTA-derived FFR involves a computational fluid dynamics simulation that utilizes adenosine-induced hyperemia in conjunction with CCTA imaging. It relies on the attenuation observed within the coronary artery, taking into account the vessel lumen’s morphology and various other parameters [40,41]. CCTA-FFR calculations are performed using a software prototype that is based on an artificial intelligence deep-machine-learning (DML) platform [41]. CCTA-FFR measurements are obtained at 10 mm proximal to MB and 10 mm distal to the MB. The change in CCTA-FFR across the MB (∆CCTA-FFR) is defined as the difference in CCTA-FFR values between the proximal and distal ends of the MB [42]. 

ΔCCTA-FFR in the systolic phase has shown the highest sensitivity and negative predictive value whereas ΔCCTA-FFR in the diastolic phase has the highest specificity among all parameters for diagnosing MB-related ischemia [43]. 

Abnormal CCTA-FFR values (defined as ≤0.80) have a prognostic role with a positive association with the symptoms of typical angina [44].

The role of CCTA-FFR has also been studied in predicting proximal plaque formation associated with LAD-MB using DML approaches [45]. While this method has been utilized for investigating MBs, further research is required to validate this strategy. 

## 5. Discussion

CCTA offers numerous advantages over invasive and other non-invasive diagnostic techniques when it comes to identifying, assessing the anatomic structure, and gauging the functional characteristics of MBs.

Its effectiveness lies in its high spatial resolution, contributing to an improved detection rate of MBs compared to coronary angiography [10,46].

CCTA facilitates precise scrutiny of the vessel wall and lumen, along with the surrounding myocardium and all adjacent structures, rendered in three dimensions. This capability permits the analysis of the artery’s trajectory and categorization of LAD-MBs based on their depth and length.

These anatomical assessments carry noteworthy clinical, prognostic, and therapeutic implications.

Notably, the deeper variant is more frequently linked to symptoms and adverse cardiac events [47,48]. MB thickness, systolic MB stenosis, and diastolic MB stenosis are independent variables predicting clinical symptoms, with diastolic MB stenosis having the highest diagnostic performance. Additionally, diastolic MB stenosis independently indicates the outcome of adverse cardiac events [49]. 

Furthermore, CCTA could assist in guiding the selection of treatment options. Medication with beta-blockers or calcium antagonists is a possible strategy. For patients resistant to medication, superficial and shorter-length MBs might be more effectively addressed through PCI. Conversely, very deep (≥5 mm) or long (≥25 mm) MBs are likely to benefit from surgical intervention [50].

Complications linked to coronary stenting encompass issues such as perforation, fracture, restenosis, and thrombosis, which have imposed limitations on its utilization [51]. Surgical methods like myotomy and unroofing can offer relief from symptoms, however, complications such as wall perforation, especially in instances of a deeply subendocardial trajectory, ventricular aneurysm formation, and post-operative bleeding, have curtailed the widespread adoption of this approach [52].

In cases of substantial MB length (≥25 mm), significant depth (≥5 mm), proximal plaques, or incomplete decompression of the bridged coronary segment during diastole, coronary artery bypass grafting (CABG) is preferred over myotomy [26]. Nevertheless, in situations involving MB, coronary blood flow exhibits dynamic behavior: it diminishes during systole and largely recovers in diastole. Consequently, during diastole post-CABG patients with MB experience competitive flow, where blood competes for passage between the coronary artery and the left internal mammary artery (LIMA). Research has demonstrated that competitive flow predominantly contributes to LIMA occlusion after CABG in MB patients [53].

A novel procedure has been devised that involves utilizing a segment of the LIMA to connect the proximal and distal portions of the MB [54]. This innovative approach exhibits better hemodynamics when compared to conventional CABG, potentially contributing to improved long-term patency.

**Figure 7:**
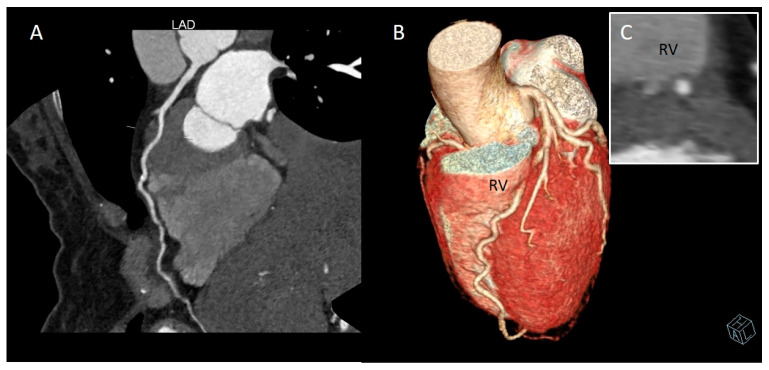
Deep myocardial bridging (MB) of left anterior descending artery (LAD), close to the right ventricle (RV) wall. (**A**) Curved multi-planar reformation of LAD. (**B**) Whole-heart volume-rendered image. (**C**) Cross-sectional image. This CCTA condition pertains to a 48-year-old woman complaining of atypical chest pain with type 2 diabetes mellitus, overweight, and smoking habit.

CCTA allows for accurate surgical pre-planning by identifying the arterial course and therefore avoiding complications such as perforation of the right ventricle wall during attempts to isolate the intramuscular artery [55,56] (Figure 7).

Although invasive coronary angiography with IVUS, dobutamine stress diastolic FFR, and instantaneous wave-free ratio (iFR) methods can reliably provide an anatomical and functional assessment of the MB [57,58,59,60], non-invasive insights into the potential hemodynamic significance of the MB may help guide the need for invasive assessment in symptomatic patients.

Specifically, CCTA has proven comparable to IVUS in determining MB length and location, the coverage of a MB by CCTA correlates well with the MB thickness by IVUS, and CCTA can give an indication of the hemodynamic significance of a MB [61].

Noninvasive imaging techniques offer potential for detecting MB and exploring myocardial ischemia in patients with MB, given that this patient group tends to be younger, exhibits fewer cardiovascular risk factors, and thus has a lower pre-test probability of obstructive coronary artery disease [62].

CCTA holds a significant role among noninvasive imaging methods within this context. CCTA may be preferred over other imaging techniques for MB identification in patients with low to intermediate risk of atherosclerotic coronary artery disease with recurrent symptoms of typical angina in order to consider dynamic ischemia from a MB in the differential diagnosis. Furthermore, CCTA may be selected for the diagnosis of MB in adults with hypertrophic cardiomyopathy because it is a noninvasive diagnostic imaging method with excellent spatial resolution. Lastly, CCTA may be of choice in athletes with exercise-induced ECG changes in whom intense physical activity increases the sympathetic tone leading to tachycardia and increased myocardial contractility, thus facilitating ischemia due to MB.

Echocardiogram contributes to the functional evaluation of MB patients, highlighting the presence of “branch steal” by revealing a localized abnormality in septal wall motion during end-systole to early-diastole, accompanied by apical sparing. This phenomenon is referred to as “septal buckling with apical sparing” [63]. Furthermore, individuals with hemodynamically significant LAD-MB exhibit reduced septal longitudinal strain compared to controls in stress echocardiographic strain imaging [64]. Nevertheless, a major limitation arises as it is not possible to ascertain whether wall motion abnormalities stem from MB, obstructive coronary artery disease, or other pathological conditions in the absence of an anatomical assessment.

Within the domain of noninvasive diagnostic techniques, cardiovascular magnetic resonance imaging (CMRI), single-photon emission computed tomography (SPECT), and positron emission tomography (PET) facilitate the quantification of myocardial blood flow both at rest and during induced hyperemia. These methods offer potential avenues for assessing inducible ischemia and coronary microvascular dysfunction in MB patients [65].

The significant drawback of CCTA is its relatively modest temporal resolution; early and mid-systolic CCTA datasets have mostly inadequate image quality due to severe motion artifacts caused by myocardial contraction. Nonetheless, suitable image quality of coronary arteries could be acquired using new generation scanners, allowing for feasible dynamic evaluation of MB.

The use of CCTA is ultimately limited by radiation and contrast exposure, however, there are now novel scanning techniques and protocols that dramatically reduce the radiation and contrast injection dosing during scanning.

## 6. Future Directions

Modern imaging technology has enabled a comprehensive assessment of both coronary anatomy and function.

Accurate detection of anatomical characteristics and hemodynamic consequences of MB is essential to evaluate its clinical significance. 

Noninvasive CCTA has been widely used to detect MB in vivo. 

Currently, there are no clinical guidelines for the diagnostic evaluation of MB, but CCTA has the prospective to play a leading role in the MB setting, having demonstrated high diagnostic performance even to detect functional ischemia in addition to an exact description and classification of anatomical features. 

Machine learning has created great potential to advance CCTA scanning, by reducing exposure to radiation and harnessing the power of artificial intelligence. The integration of artificial intelligence algorithms into CCTA systems will enable physicians to provide better images, who will in turn provide better treatments based on more accurate diagnosis.

## 7. Conclusions

MB is a common congenital anomaly encountered frequently in clinical practice. For quite some time, MB was regarded as an incidental discovery. Yet, an increasing volume of evidence points to its connection with symptoms and its potential to result in unfavorable cardiac events.

Thanks to advances in CCTA technology, we now have a powerful noninvasive method to evaluate the location, depth, and length of a MB and any concomitant atherosclerotic changes. Furthermore, functional assessment of MB with CCTA is proving of great interest. 

Future development of CCTA approaches dedicated to the evaluation of MB is warranted for considering CCTA the tool of choice of the diagnostic and prognostic evaluation of MB.

## Data Availability

No new data were created in this study.
